# High-Degree Posterior Rotational Osteotomy Provides Post-operative Stability Superior to Anterior Rotational Osteotomy for Non-traumatic Osteonecrosis of the Femoral Head: Evaluation Using Computed Tomography

**DOI:** 10.7759/cureus.76125

**Published:** 2024-12-21

**Authors:** Satoe Tanabe, Takashi Atsumi, Masanori Nishi, Yasushi Yoshikawa, Ryosuke Nakanishi, Minoru Watanabe, Tsubasa Ishikawa, Yuki Usui, Koji Kanzaki, Yoshifumi Kudo

**Affiliations:** 1 Orthopaedic Surgery, Showa University Koto Toyosu Hospital, Tokyo, JPN; 2 Orthopaedic Surgery, Showa University Fujigaoka Hospital, Yokohama, JPN; 3 Orthopaedic Surgery, Showa University School of Medicine, Tokyo, JPN; 4 Orthopaedic Surgery, Sassa General Hospital, Tokyo, JPN; 5 Orthodontics, Showa University Fujigaoka Hospital, Yokohama, JPN

**Keywords:** anterior rotational osteotomy, computed tomography, high-degree posterior rotational osteotomy, instability of the hip joint, nontraumatic osteonecrosis of the femoral head

## Abstract

Introduction

This study evaluated hip joint dynamic instability in patients with non-traumatic osteonecrosis of the femoral head (ONFH) with extensive lesions, who had undergone anterior rotational osteotomy (ARO) and high-degree posterior rotational osteotomy (HDPRO), based on the femoral head translation observed by computed tomography (CT) at 0° and 45° hip flexion.

Materials and methods

Medical records of patients who had undergone transtrochanteric rotational osteotomy for non-traumatic ONFH were retrospectively reviewed to identify patients who had undergone CT examinations six weeks post-operatively. In all, 64 hips (60 patients; 19 men and 41 women), comprising 36 hips treated with HDPRO and 28 hips treated with ARO, respectively, were included. The difference in the distance from the femoral head to the acetabulum between images acquired in the neutral position and those acquired in the 45° flexion position was measured. Femoral head translation of >1 mm between the neutral position and 45° hip flexion was defined as instability.

Results

Hip joint instability was observed in 14% (5/36) and 25% (7/28) of hips treated with HDPRO and ARO, respectively, at all disease stages. Instability was observed significantly less frequently in the hips treated with HDPRO (9%, or 2/23), compared with those that underwent ARO (50%, or 6/12) in the advanced stages (no joint space narrowing and femoral head collapse of ≥3 mm) (p = 0.01).

Conclusion

HDPRO yields post-operative stability, even in patients with advanced femoral head collapse. Thus, it is a valuable option for the management of non-traumatic ONFH with extensive lesions.

## Introduction

Non-traumatic osteonecrosis of the femoral head (ONFH), an avascular necrosis observed in the femoral head in association with steroid administration and alcohol abuse, occurs frequently in young adults [1]. The extent of necrosis in the loading portion of the acetabulum drives the progression of femoral head collapse, which can lead to hip joint destruction at an early stage [2]. Hip arthroplasty is often indicated in the long term; however, the mid-to-long-term outcomes tend to be unfavorable in younger patients [3,4]. Therefore, various joint-preserving surgical approaches have been reported to preserve the hip joint in young patients [5-19].

Core decompression [5-7] and vascularized bone grafting [8-10] have demonstrated favorable outcomes in cases with minimal necrosis without collapse, but poor outcomes in cases with advanced collapse [5-10]. Varus osteotomy of the femoral head is effective in cases with a viable lesion on the lateral side of the acetabulum [11,12]; however, its success rate is low in cases with collapse [12].

Anterior rotational osteotomy (ARO), a joint-preserving surgical technique developed by Sugioka for patients with significant necrosis [13], preserves the joint by rotating and transposing the remaining viable lesion on the posterior femoral head to a weight-bearing area of the acetabulum to bear the load [13-15]. ARO is indicated in cases with one-third or more of the viable lesion present on the posterior femoral head; however, few cases satisfy this criterion.

High-degree posterior rotational osteotomy (HDPRO) was introduced by Atsumi and Kuroki [16] for cases with a viable lesion located in the antero-inferior part of the femoral head, despite extensive necrosis of the weight-bearing area, and for cases with one-third or less of the viable lesion present posteriorly, for which ARO is contraindicated. They reported favorable outcomes and the occurrence of remodeling following HDPRO [17-21].

The viable lesion on the anterior femoral head is transposed to the weight-bearing area of the acetabulum to bear the weight after HDPRO; consequently, the femoral head is always placed in a stable condition within the acetabulum during activities of daily living, wherein flexion is the main movement, with the viable lesion positioned anteriorly [16-20]. In contrast, necrotic lesions are positioned anteriorly following ARO; consequently, the collapsed and necrotic lesion is transposed to the weight-bearing area of the acetabulum during flexion of the hip joint, rendering the anterior hip joint unstable [22]. It was hypothesized that a difference would be observed in the stability of the hip joint within the acetabulum following ARO and HDPRO in patients with advanced collapse. Therefore, this study aimed to evaluate the dynamic instability of the hip joint following ARO and HDPRO for ONFH, using horizontal computed tomography (CT) images acquired in the neutral position and at 45° hip flexion in the supine position.

## Materials and methods

Patients' characteristics

The medical records of patients who had undergone transtrochanteric rotational osteotomy for non-traumatic ONFH at our institution were retrospectively reviewed to identify patients who underwent CT examination of the hip six weeks post-operatively. Overall, 68 hips from 64 patients were included in this study. Four hips from four patients with severe collapse were excluded owing to the difficulty in identifying the posterior articular surface of the femoral head for measurement. Thus, 64 hips from 60 patients (41 male and 19 female; mean age at surgery, 34.5 years (range, 17-57)) were finally included in the analysis. Further, 23 hips from 23 patients were unilaterally affected; thus, the unaffected side was used as the control. These unaffected 23 hips were asymptomatic, with no findings indicating necrosis on plain radiography or magnetic resonance imaging (MRI).

A total of 1, 52, and 11 hips were classified as having Stage II, Stage III, and Stage IV disease, respectively, according to the Association Research Circulation Osseous Classification [23]. Similarly, 1, 17, 35, and 11 hips were classified as having Stage II (no collapse of the femoral head), Stage IIIA (collapse of the femoral head ≤3 mm), Stage IIIB (collapse of the femoral head ≥3 mm), and Stage IV (joint space narrowing) disease, respectively, according to the Japanese Investigation Committee classification [24]. Based on the extent of necrosis in the loading portion of the joint, 3, 22, and 39 hips were classified as having Type B (intact area present on the lateral two-thirds of the loading surface), Type C1 (intact area present on the lateral one-third of the loading surface), and Type C2 (no intact area present on the loading surface) disease, respectively. Significant differences were not observed in terms of the pre-operative stage and type following ARO and HDPRO (p = 0.22 and p = 0.51, respectively) (Table 1). Triggers for necrosis included heavy steroid administration, alcohol abuse, and no evident trigger in 25 (28 hips), 30 (31 hips), and 5 (5 hips) patients, respectively. The underlying diseases necessitating steroid administration were Behçet’s disease, facial palsy, ulcerative colitis, interstitial pneumonia, asthma, pituitary tumor, and germinoma in one case each; systemic lupus erythematosus in five cases; nephropathic syndrome in four cases; idiopathic thrombocytopenic purpura in three cases; and mixed connective tissue disease, ophthalmologic disease, and sudden hearing loss in two cases each. Among the 60 participants, bilateral and unilateral hips were affected in 37 and 23 patients, respectively. Among the 37 patients with bilaterally affected hips, seven underwent HDPRO (n = 3), ARO (n = 3), or total hip arthroplasty (THA; n = 1) on both sides. The remaining 30 patients underwent surgery on one side, as the contralateral side was asymptomatic with a minimal necrotic lesion and no collapse; these cases required observation only. The three participants who underwent HDPRO and the patients who underwent ARO were included in this study.

**Table 1:** Patients' characteristics. * denotes the Japanese Investigation Committee classification. ARO: Anterior rotational osteotomy; HDPRO: High-degree posterior rotational osteotomy; ONFH: Osteonecrosis of the femoral head

Characteristics	ARO	HDPRO
No. of patients/hips	27/28	33/36
Sex, male N (%)	20 (74)	21 (64)
Mean age at surgery (range) (years)	38.9 (23-56)	31.1 (17-48)
Etiology of ONFH (hips)		
Steroid (%)	9 (32)	19 (53)
Alcohol (%)	19 (68)	12 (33)
No risk factor (%)	0 (0)	5 (14)
Stage* (hips)		
II	1	0
IIIA	10	7
IIIB	12	23
IV	5	6
Type* (hips)		
B	2	1
C1	11	11
C2	15	24
Mean rotation angle (range), (degree)	77.3 (70-90), anterior	113.2 (110-140), posterior

Indication and post-operative management

ARO was performed when the intact area of the posterior part of the femoral head was one-third or more. HDPRO was performed in cases where the area from the anterior to the anteroinferior part of the femoral head was intact, and when ARO was contraindicated. We assessed the intact area of the femoral head using anteroposterior, 90° and 130° flexion lateral radiographic views, along with an MRI of the hip joint [15-18]. Overall, 28 (27 patients) and 36 (33 patients) hips underwent ARO and HDPRO, respectively. The mean angle of anterior rotation, mean posterior rotational angle, and mean varus angle were 77.3° (range, 70-90°), 113.2° (range, 110-140°), and 18.3° (range, 10-25°), respectively. All surgeries were performed by a single surgeon (TA) (Table 1).

The proprietary F-system (MIZUHO, Tokyo, Japan) was used to secure the fixation of the transtrochanteric osteotomy in all cases [16,18].

All patients used a wheelchair and commenced motion exercises one week after ARO and HDPRO. Partial weight-bearing was initiated four to five weeks post-operatively in both groups.

CT analysis

CT examinations were conducted using Light Speed Plus (GE Yokogawa Medical Systems, Inc., Tokyo, Japan) six weeks post-operatively, a timeframe conducive to obtaining images of the intended positions. This duration facilitated joint stabilization following the procedure, which involved articular capsule ring incision and muscle release.

**Figure 1:**
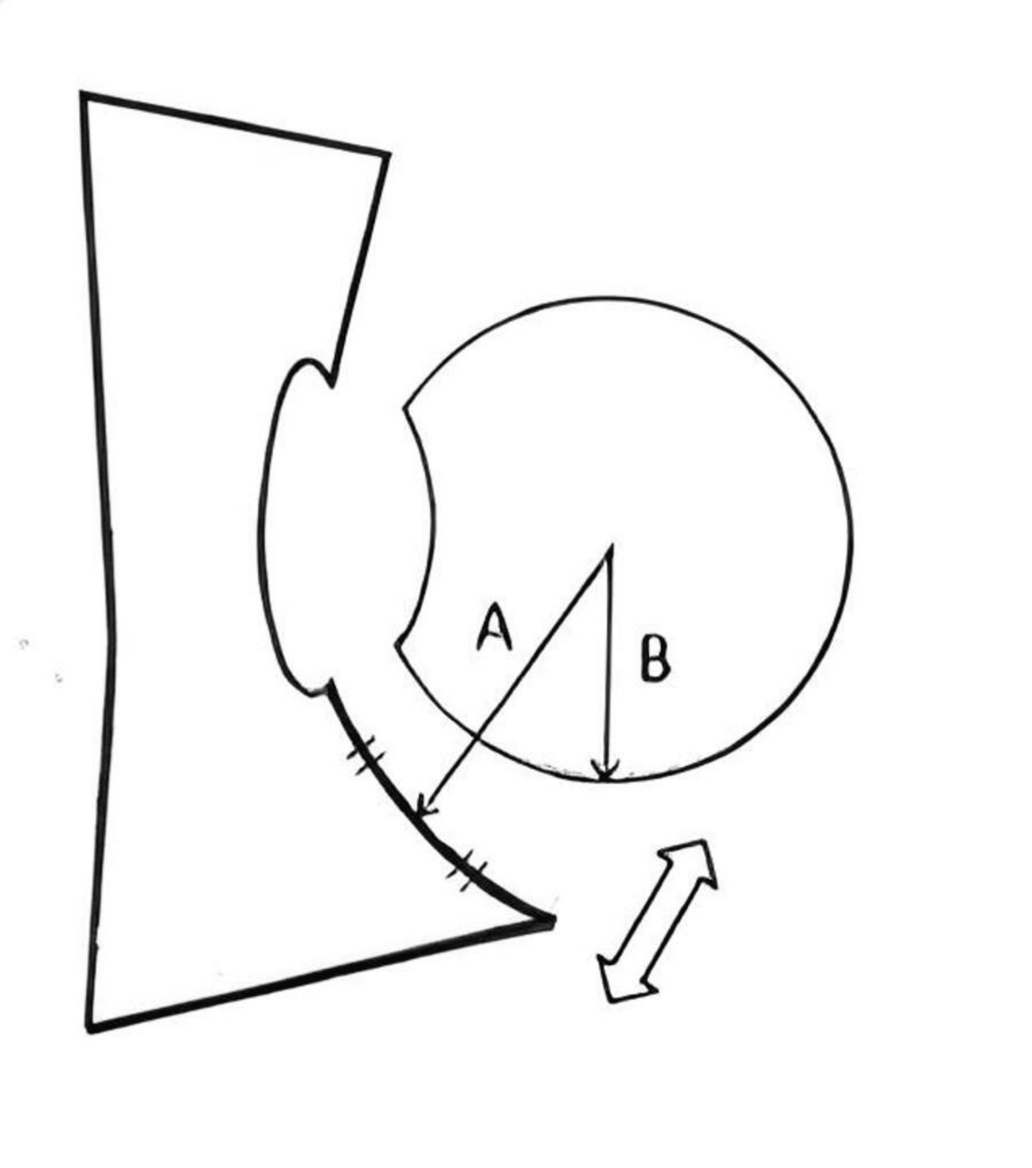
The distance from the posterior acetabular joint surface to the posterior joint surface of the femoral head (two-way arrow) was measured as the difference between the distance from the middle of the posterior acetabular roof to the center of the femoral head (arrow A), and the radius of the femoral head (arrow B). Image credit: Satoe Tanabe

**Figure 2:**
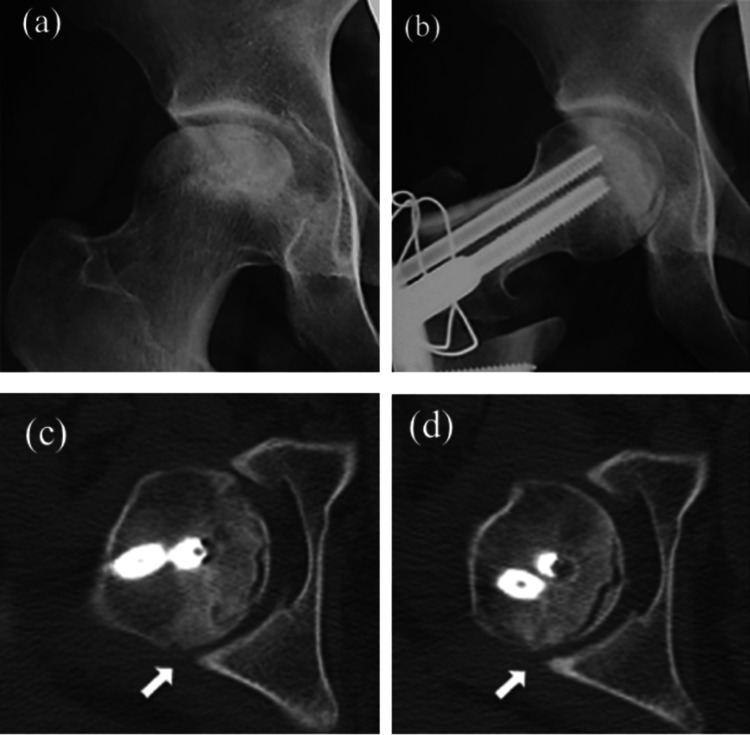
Diagnostic images and CT images of a 44-year-old male. (a) Diagnostic images of a 44-year-old male with a history of sudden hearing loss treated with high-dose corticosteroids. The pre-operative anteroposterior radiograph of the right hip shows a severely collapsed lesion and no viable area of the loaded portion. (b) A 110° posterior rotational osteotomy with 20° varization was performed. (c) CT images were acquired six weeks post-operatively. The distance from the posterior acetabular joint surface to the posterior joint surface of the femoral head in the neutral position is 3.52 mm (white arrow) on the horizontal CT image. (d) The distance from the posterior acetabular joint surface to the posterior joint surface of the femoral head in the 45° flexion position is 3.79 mm (white arrow) on the horizontal CT image. The translation of the femoral head between the neutral and 45° flexion positions is 0.27 mm, which is considered stable. CT: Computed tomography

**Figure 3:**
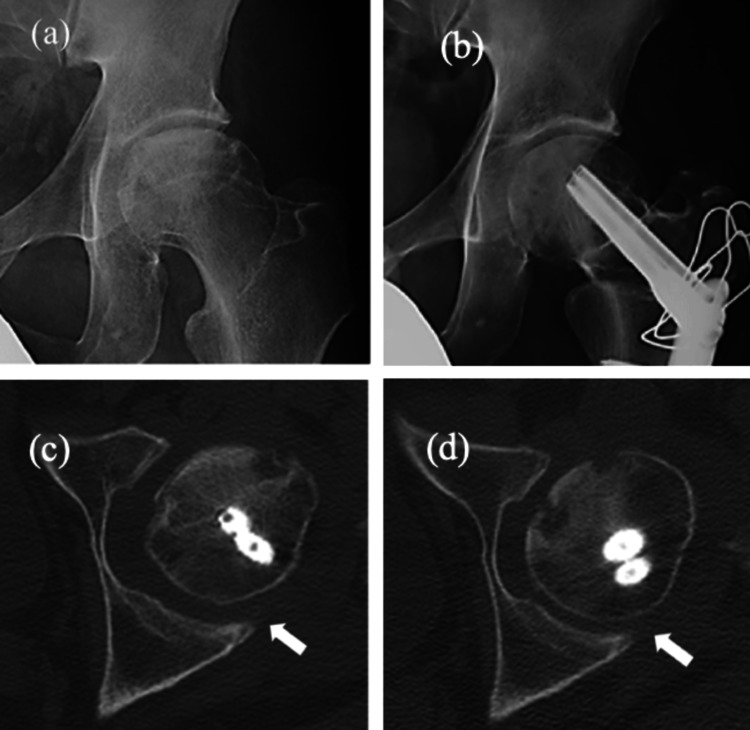
Diagnostic images and CT images of a 37-year-old male. (a) Diagnostic images of a 37-year-old male with alcohol-related osteonecrosis of the femoral head. The pre-operative anteroposterior radiograph of the left hip shows a severely collapsed lesion with no viable area in the loaded portion. (b) A 70º anterior rotational osteotomy with 25º varization was performed. (c) CT images were acquired six weeks post-operatively. The distance from the posterior acetabular joint surface to the posterior joint surface of the femoral head in the neutral position is 6.33 mm (white arrow) on the horizontal CT image. (d) The distance from the posterior acetabular joint surface to the posterior joint surface of the femoral head in the 45° flexion position is 3.81 mm (white arrow) on the horizontal CT image. The translation of the femoral head between the neutral and 45º flexion positions is 2.52 mm, indicating instability. CT: Computed tomography

CT images were acquired with the patient in the supine position, whereas hip images were obtained in the neutral (0° flexion) and 45° flexion positions, without hip rotation or weight-bearing. The distance from the posterior acetabular joint surface to the posterior joint surface of the femoral head was measured on the horizontal CT images (Figures 1-3).

**Figure 4:**
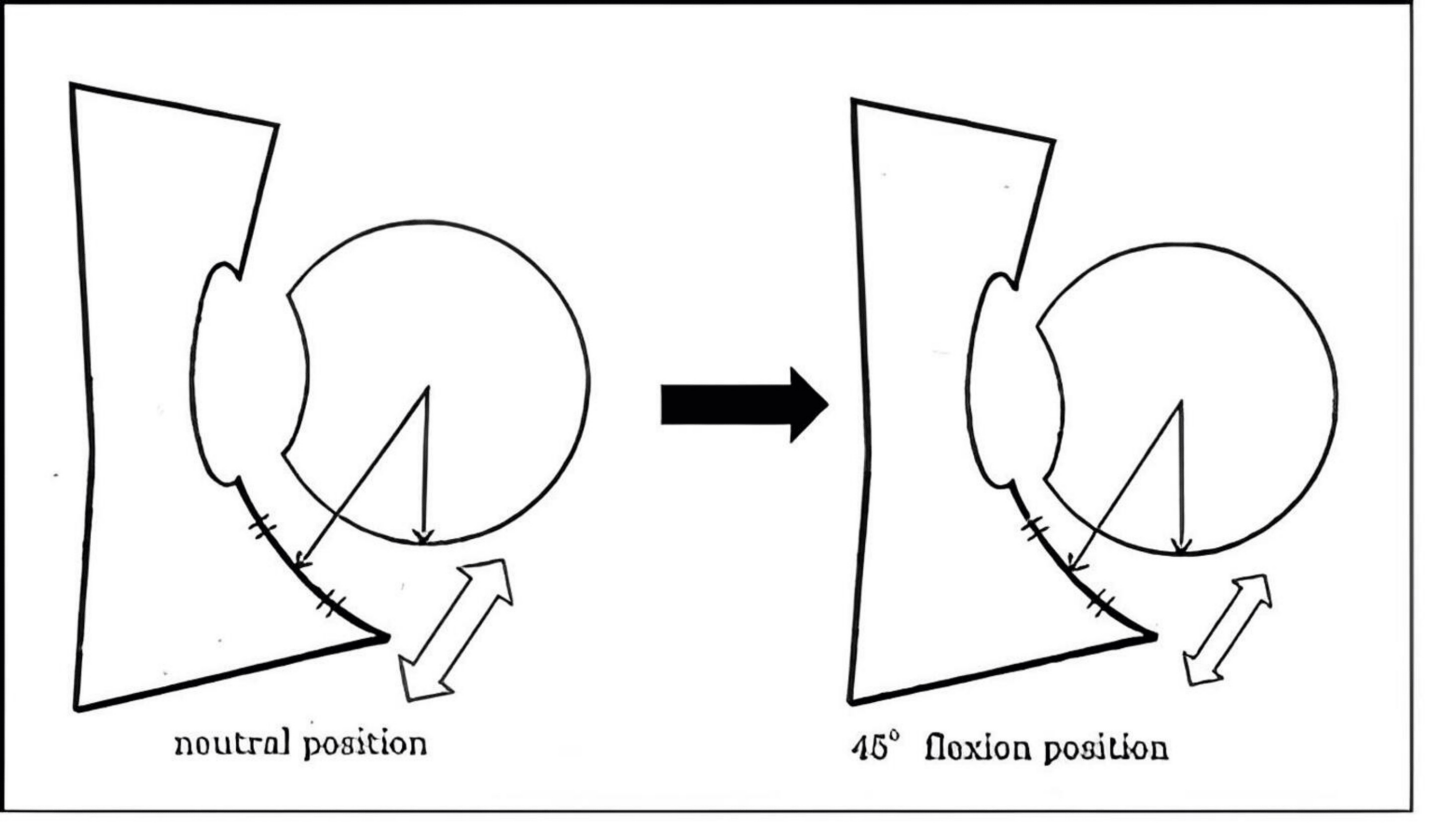
Instability was estimated by comparing the distance in the neutral position and that in the 45° flexion position. Instability was defined as a difference of >1.0 mm. Image credit: Satoe Tanabe

The difference in the distance from the femoral head to the acetabulum between images acquired in the neutral position and those acquired in the 45° flexion position was measured. Instability was defined as a difference of >1.0 mm (Figures 2-4). The measurements were performed three times by the same orthopedic surgeon (ST) using FUJIFILM SYMAPSE (Fujifilm Medical Co., Ltd., Tokyo, Japan), and the average values were considered the actual measured values.

The following items were examined and analyzed: first, the translation of the femoral head on the unaffected side of the hip joint was investigated. Additionally, the study compared the translation of the femoral head between the ARO and HDPRO groups. The analysis was further expanded to include comparisons of the translation of the femoral head between these groups, according to disease stage and disease type.

Statistical analysis

The relationship among hip instability, operative procedure, and stage or type of disease was analyzed via Fisher’s exact probability test. Cases with a hazard value of ≤0.05 were considered to show a statistically significant difference. All statistical analyses were performed using JMP Pro 17 (SAS Institute, Cary, NC, USA).

## Results

Femoral head translation on the unaffected side

The mean translation of the femoral head was 0.14 mm (range, 0.00-0.39 mm) in the 23 unaffected hips, with none exceeding 1 mm.

Comparison of instability following ARO and HDPRO

Seven (25%) of the 28 hips treated with ARO, and five of the 36 hips (14%) treated with HDPRO exhibited instability. No significant differences were observed between the two groups in terms of instability (p = 0.33) (Table 2).

**Table 2:** Post-operative instability following ARO and HDPRO. § denotes no significant difference (p = 0.33). ARO: Anterior rotational osteotomy; HDPRO: High-degree posterior rotational osteotomy

Variable	Stability	Instability	Total
ARO, N (%)	21 hips (75)	7 hips (25)^§^	28 hips
Translation distance, mean (range) (mm)	0.40 (0.02-0.95)	2.18 (1.33-3.25)	0.84 (0.02-3.25)
HDPRO, N (%)	31 hips (86)	5 hips (14)^§^	36 hips
Translation distance, mean (range) (mm)	0.35 (0.02-0.85)	1.69 (1.07-2.29)	0.54 (0.02-2.29)

Examination of instability according to disease stage

One hip with Stage II (no collapse of the femoral head) disease exhibited no instability after ARO. One (10%) of the 10 hips with Stage IIIA (collapse of the femoral head ≤3 mm) disease exhibited instability after ARO; two (29%) of the seven hips exhibited instability after HDPRO. With regard to post-operative instability observed in patients with Stage IIIA disease (p = 0.53), no significant differences were observed between the two groups. Six (50%) of the 12 hips with Stage IIIB (collapse of the femoral head ≥3 mm) disease exhibited instability after ARO (Figures 4a-4d); two (9%) of the 23 hips exhibited instability after HDPRO. The remaining 21 hips in the HDPRO group did not exhibit instability (Figures 3a-3d).

A significant difference was observed between groups in terms of instability among patients with Stage IIIB disease (p = 0.01). Five hips with Stage IV (joint space narrowing) disease exhibited no instability after ARO; one of the six hips (17%) exhibited instability after HDPRO (Table 3).

**Table 3:** Post-operative instability following ARO and HDPRO according to pre-operative disease stage. † denotes no significant difference (p = 0.53); * denotes significant difference (p = 0.01). ARO: Anterior rotational osteotomy; HDPRO: High-degree posterior rotational osteotomy

Variable	Stage IIIA	Stage IIIB	Stage IV
Instability after ARO, N (%)	1 of 10 hips (10)^†^	6 of 12 hips (50)*	0
Translation distance, mean (range) (mm)	1.63	2.29 (1.33-3.25)	Not applicable
Instability after HDPRO, N (%)	2 of 7 hips (29)^†^	2 of 23 hips (9)*	1 of 6 hips (17)
Translation distance, mean (range) (mm)	1.68 (1.07-2.29)	1.62 (1.17-2.08)	1.86

Examination of instability according to disease type

Two hips with Type B (intact area present on the lateral two-thirds of the loading surface) disease exhibited no instability after ARO; one hip exhibited instability after HDPRO.

Two (18%) of the 11 hips with Type C1 (intact area present on the lateral one-third of the loading surface) disease exhibited instability after ARO; two (18%) of the 11 hips exhibited instability after HDPRO. Five (33%) of the 15 hips with Type C2 (no intact area present on the loading surface) disease exhibited instability after ARO (Figure 3); two (8%) of the 24 hips exhibited instability after HDPRO. The remaining 22 hips treated with HDPRO exhibited no instability (Figure 2). No significant differences were observed between groups in terms of instability of hips with Type C2 disease (p = 0.08) (Table 4).

**Table 4:** Post-operative instability following ARO and HDPRO according to pre-operative disease type. § denotes no significant difference (p = 0.08). ARO: Anterior rotational osteotomy; HDPRO: High-degree posterior rotational osteotomy

Variable	Type B	Type C1	Type C2
Instability after ARO, N (%)	0	2 of 11 hips (11)	5 of 15 hips (33)^§^
Translation distance, mean (range) (mm)	Not applicable	1.48 (1.33-1.63)	2.46 (1.63-3.25)
Instability after HDPRO, N (%)	1 of 1 hip	2 of 11 hips (18)	2 of 24 hips (8)^§^
Translation distance, mean (range) (mm)	2.29	1.46 (1.07-1.86)	1.62 (1.17-2.08)

Five (71%) of the seven hips with Type C2 Stage IIIB (no intact area present on the loading surface and collapse of the femoral head ≥3 mm) disease exhibited instability after ARO (Figure 3); two (11%) of the 19 hips exhibited instability after HDPRO. The remaining 17 hips treated with HDPRO exhibited no instability (Figure 2). Significant differences were observed between groups in terms of the number of hips with Type C2 Stage IIIB disease exhibiting instability (p < 0.01) (Table 5).

**Table 5:** Post-operative instability following ARO and HDPRO in patients with Type C2 Stage IIIB (with no intact area present on the loading surface and collapse of the femoral head ≥3mm). * denotes significant difference (p < 0.01). ARO: Anterior rotational osteotomy; HDPRO: High-degree posterior rotational osteotomy

Variable	Stability	Instability	Total
ARO, N (%)	2 hips (29)	5 hips (71)*	7 hips
Translation distance, mean (range) (mm)	0.36 (0.11-0.62)	2.46 (1.94-3.25)	1.86 (0.11-3.25)
HDPRO, N (%)	17 hips (89)	2 hips (11)*	19 hips
Translation distance, mean (range) (mm)	0.43 (0.02-0.85)	1.62 (1.17-2.08)	0.59 (0.02-2.08)

## Discussion

Previous reports have described the natural course of ONFH. Nishii et al. reported that the progression of collapse was not observed in cases with ≤2 mm collapse and the intact area occupying the lateral one-third or less of the weight-bearing area; surgical intervention is not indicated for such cases [25]. Ohzono et al. reported early progression of collapse during the natural course in cases with extensive necrosis [2]. Thus, conservative therapy in cases with no progression of collapse is effective in a limited number of cases, and some type of surgical treatment is inevitable in the near future for cases with a substantial amount of necrosis. Unfavorable mid-to-long-term outcomes of THA have been observed in young patients with ONFH [3,4]; consequently, joint-preserving surgery is the preferred choice for these patients.

Sugioka et al. [14] reported that ARO yielded a good post-operative outcome in 93% of cases with ≥36% of the intact loading area available; however, the outcome was poor in cases with advanced collapse of ≥2 mm. Sugano et al. reported no post-operative collapse in the mid-to-long-term following ARO in cases with up to Stage II disease, as per the Ficat classification [26]; however, collapse was observed in 30% of Stage III cases [27]. Inao et al. reported that a collapse of ≤2 mm yielded favorable long-term outcomes following ARO, despite the presence of collapse. In contrast, cases with >2 mm collapse or arthritic changes exhibited poor clinical outcomes and imaging findings [28]. However, Atsumi et al. revealed that remodeling occurred in the necrotic region that was transposed to the non-weight-bearing lesion and that a spherical femoral head was formed after HDPRO, even in cases with collapse and extensive necrosis [19]. The findings of this study suggest that patients with Stage IIIB disease and significant collapse are significantly less susceptible to instability following HDPRO. HDPRO is more resistant to instability, even in cases with severe collapse, owing to the following reasons: first, the necrotic lesion is positioned on the postero-medial side from the medial side of the acetabulum following HDPRO, whereas the spherical viable lesion is positioned on the anterior part of the femoral head; second, the femoral head remains in a stable condition in the acetabulum as the good viable lesion on the anterior femoral head is transposed to the loading portion of the acetabulum and bears weight in the flexion position, which is the core movement in activities of daily living [16-18]. Moreover, the necrotic lesion moves further away from the weight-bearing lesion during flexion, leading to increased stability. Atsumi et al. reported that the extent of the intact area of the femoral head present in the loading portion of the acetabulum after HDPRO remained unchanged between the 0° and 45° flexion positions on the antero-posterior radiographs of the hip joint [18].

The posterior column artery feeding the femoral head is markedly stretched out as a result of rotation following ARO. Thus, blood supply in the articular capsule passage may be impaired if rotational manipulation is performed in a rough manner intra-operatively. In contrast, the posterior column artery is transferred medially without stretching following HDPRO, owing to rotation; thus, a rotation of ≥100° is possible [16]. This may also contribute to the effectiveness of HDPRO in cases with significant collapse and necrosis.

HDPRO is technically demanding [29]; however, it is indicated even in cases with a significant amount of necrosis. Moreover, a sufficient amount of intact area is obtained in weight-bearing lesions of the acetabulum post-operatively, and remodeling is possible even in advanced-stage cases [19].

Furthermore, weight distribution is facilitated in a stable manner post-operatively, and the repair of the necrotic region is promoted at an early stage, as instability is unlikely to occur after the HDPRO procedure.

This study has certain limitations. First, it was a retrospective study with a limited number of patients. Further large-scale studies must be conducted to validate these findings. Second, this study assessed instability using early post-operative CT scans; however, it did not evaluate the impact of post-operative remodeling or the correlation with post-operative outcomes. However, joint stability is a pivotal factor influencing long-term joint preservation. Moreover, joint instability affects the progression of joint destruction [30]. Attaining stability during the early post-operative period facilitates favorable remodeling after HDPRO. Furthermore, the long-term outcomes of HDPRO have demonstrated a potential correlation, indicating its relevance, thereby warranting further investigation [20].

## Conclusions

In conclusion, HDPRO is more resistant to post-operative joint instability than ARO in cases with advanced collapse. The difference in post-operative localization of the necrotic area between ARO and HDPRO can affect joint instability. Thus, HDPRO is an effective joint-preserving surgery, even in cases of ONFH with advanced collapse, with long-term efficacy in terms of joint preservation.
